# Biomechanical analysis of different techniques for residual bone defect from tibial plateau bone cyst in total knee arthroplasty

**DOI:** 10.3389/fbioe.2024.1498882

**Published:** 2024-10-30

**Authors:** Dehua Liu, Zhuang Miao, Wenfei Zhang, Chuanwen Liu, Longzhuo Du, Yuanlong Zhu, Yange Luo, Weibo Zheng, Jianli Zhou, Peilai Liu, Xuezhou Li, Ming Li

**Affiliations:** ^1^ Department of Orthopaedics, Qilu Hospital, Cheeloo College of Medicine, Shandong University, Jinan, Shandong, China; ^2^ Key Laboratory of Ultra-Weak Magnetic Field Measurement Technology, Ministry of Education, School of Instrumentation and Optoelectronic Engineering, Beihang University, Beijing, China; ^3^ Psychological Department, Qilu Hospital of Shandong University Dezhou Hospital, Dezhou, Shandong, China; ^4^ Department of Orthopaedics, Qilu Hospital of Shandong University Dezhou Hospital, Dezhou, Shandong, China; ^5^ Nuclear Medicine Department, Qilu Hospital of Shandong University Dezhou Hospital, Dezhou, Shandong, China

**Keywords:** bone defect, bone cyst, finite-element analysis, biomechanical test, total knee arthroplasty, 3D printing technology

## Abstract

**Background:**

In patients with tibial plateau bone cysts undergoing total knee arthroplasty (TKA), bone defects commonly occur following tibial plateau resection. Current strategies for addressing these defects include bone grafting, bone cement filling, and the cement-screw technique. However, there remains no consensus on the optimal approach to achieve the best surgical outcomes. This study aims to evaluate the most effective repair method for residual bone defects following tibial plateau bone cyst repair during TKA from a biomechanical perspective.

**Methods:**

The treatment options for tibial plateau bone defects were classified into four categories: no treatment, cancellous bone filling, bone cement filling, and the cement-screw technique. Finite-element analysis (FEA) was employed to evaluate stress distribution and displacement across the models for each treatment group. In addition, static compression mechanical tests were used to assess the displacement of the models within each group.

**Results:**

FEA results indicate that when employing the cement-screw technique to repair tibial plateau bone defects, the maximum stress on the prosthesis and the cement below the prosthesis is minimized, while the maximum stress on the cancellous bone is maximized. And the displacement of each component is minimized. Biomechanical tests results further demonstrate that the displacement of the model is minimized when utilizing the cement-screw technique for tibial plateau bone defects.

**Conclusion:**

Using cement-screw technique in treating residual tibial bone defects due to bone cysts in TKA offers optimal biomechanical advantages.

## 1 Introduction

Total knee arthroplasty (TKA) is one of the most effective surgical interventions for relieving pain and restoring function of knee in patients with advanced osteoarthritis (Cushnaghan et al., 2009; Carr et al., 2012; Price et al., 2018). Due to prolonged degenerative changes and weight-bearing wear, tibial plateau bone cysts are frequently observed during TKA procedures (Burnett et al., 2019; Tanamas et al., 2010). Residual bone defects from these cysts, following tibial plateau resection, can hinder both anatomical reconstruction and functional recovery of the knee joint (Lei et al., 2019; Lombardi et al., 2010).

The widely utilized Anderson Orthopaedic Research Institute (AORI) classification system categorizes knee-related bone defects into three types based on their size and location (Bieganowski et al., 2022; Sheth et al., 2017). Type I defects are characterized by mild bone loss with intact metaphyseal ends and are generally amenable to correction using standard TKA procedures. Type III defects involve extensive damage to the femoral condyles or tibial plateaus, often requiring customized components to effectively address the defect. Type II defects involve metaphyseal bone loss and are further subdivided into subtypes A and B: subtype A affects either the femoral condyles or tibial plateaus, while subtype B involves both. For Type II bone defects, treatment options typically include the cement-screw technique, bone cement filling, and bone grafting (Aggarwal and Baburaj, 2021; Shafaghi et al., 2019). However, a consensus on the optimal surgical approach for addressing bone defects resulting from tibial plateau bone cysts has yet to be established.

Cement filling, bone grafting, and the cement-screw technique are all effective methods for managing Type 2A bone defects, accommodating a range of defect sizes and configurations. Cement filling involves packing cement into the bone defect to enhance stability between the defect area, surrounding tissue, and the implant through adhesion (Qiu et al., 2012). Bone grafting, which includes both autografts and allografts, primarily uses cancellous bone to fill the defect, with the main difference being the source of the graft material (Lotke et al., 2006; Bauman et al., 2009; Wang et al., 2013). The cement-screw technique further augments fixation strength at the defect site by placing screws within the cement; however, precise screw placement is critical to avoid compromising the positioning of the prosthesis (Berend et al., 2014; Özcan et al., 2021; Berend et al., 2015).

Finite-element analysis (FEA) and biomechanical tests are widely utilized methodologies for investigating the biomechanics of orthopaedic implants (Campbell and Glüer, 2017; Lewis et al., 2021; Beitler et al., 2022). FEA provides accurate simulations by modeling bone structures (Siddiqi et al., 2021), while biomechanical tests involve measuring and analyzing experimental data to obtain quantitative insights into the mechanical behavior of skeletal systems (Adames et al., 2023; Schwer et al., 2024). These methods complement each other, with biomechanical tests helping to assess the mechanical properties of bone tissue and validate FEA results (Liu et al., 2022; Pei et al., 2023). Additionally, we have developed a standardized surgical guide to create an anteromedial tibial encapsulated bone defect, ensuring consistency and precision in biomechanical test models.

In this study, we combined FEA and biomechanical tests to explore the biomechanical differences among cement filling, cancellous bone filling, and the cement-screw technique for repairing tibial plateau bone defect in total TKA. Additionally, we compared these methods against a model that did not receive any specific treatment for the bone defect.

## 2 Methods

### 2.1 Statistical analysis of tibial plateau bone cyst regions in patients treated with TKA

**FIGURE 1:**
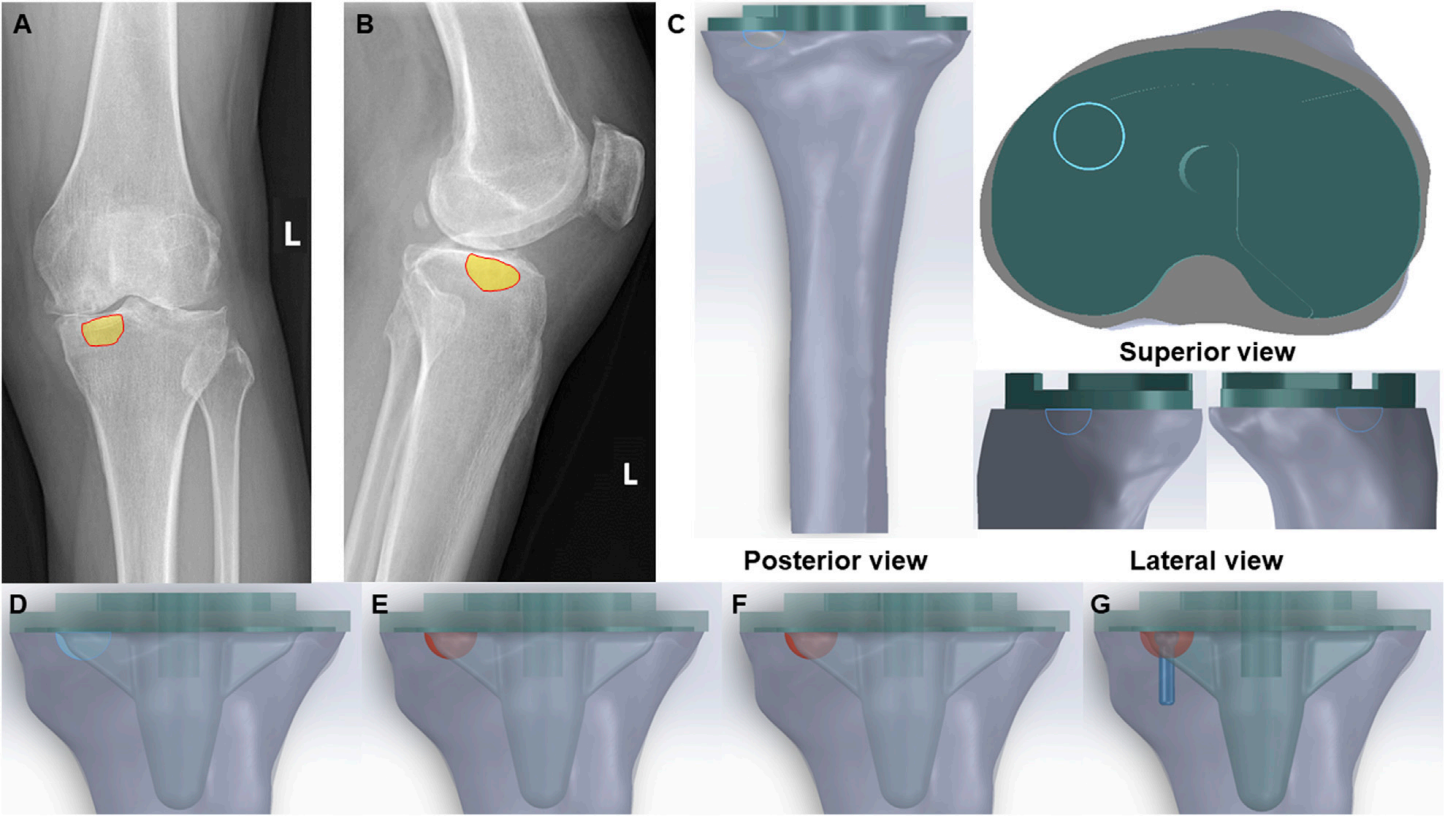
Clinical scenarios and FE models of bone cyst. **(A)** Anteroposterior radiograph of a bone cyst case (Yellow part-bone cyst defect). **(B)** Lateral radiograph of a bone cyst case (Yellow part-bone cyst defect). **(C)** Posterior view, superior view and lateral view of the integral FE model of bone cyst defect model (Green part-prothesis and blue hemisphere-bone cyst defect). **(D)** Bone cyst defect without filling group (Blue hemisphere-untreated bone cyst defect). **(E)** Bone cyst defect filled with cancellous bone group (Red hemisphere-treated bone cyst defect). **(F)** Bone cyst defect filled with cement group (Red hemisphere-treated bone cyst defect). **(G)** Bone cyst defect filled with cement-screw technique group (Red hemisphere-treated bone cyst defect and blue part-screw).

We collected preoperative X-ray data from 31 patients who underwent TKA at Qilu Hospital of Shandong University Dezhou Hospital. The preoperative X-rays identified tibial plateau bone cysts (Figures 1A, B). A statistical analysis was conducted to assess the distribution and size of these tibial plateau bone cysts.

### 2.2 Finite-element model

The CT images of the tibia from a normal adult male, weighing 65 kg, were imported into Mimics 21.0 (Materialise, Belgium). The bone tissue was identified and extracted by the gray value of the tissue, and then the three-dimensional (3D) model of the tibia was constructed by using the region segmentation command to create a 3D model of the tibia. Additionally, a tibial prosthesis (Depuy Synthes Attune, Massachusetts, United States) was scanned with a Sinscan 3D scanner (System Ceramics, Italy) and imported in STL format into Geomagic Studio 2012 (Geomagic, United States) for model smoothing.

The components of the finite-element models were imported into SolidWorks 2022 (Dassault Systems SolidWorks Corp, United States), and the tibial model was resected following standard surgical procedures. Initially, the tibial model was resected 8 mm below the lateral tibial plateau articular surface, perpendicular to the mechanical axis. Subsequently, based on clinical data, additional resection was carried out on the anteromedial tibial plateau to create a bone defect with a depth of 6 mm and a circular surface radius of 6 mm. To facilitate optimal fixation for biomechanical tests, the tibia was further sectioned perpendicular to the mechanical axis at a distance of 17 cm from the tibial plateau.

Subsequently, bone defects were addressed using three techniques: cancellous bone filling, cement filling, and the cement-screw technique, with prosthetic components assembled accordingly according to standard TKA procedures. Besides, there is a volume gap between the bottom surface of the prosthesis and the tibial plateau in TKA, which fills the bone cement. To optimize the cement-screw technique and ensure proper implantation, a screw was vertically inserted into the central area of the bone defect, perpendicular to the tibial plateau surface. The screw employed in this study was a standard 3.5 mm diameter by 16 mm length cancellous bone screw, modeled in SolidWorks 2022. The complete assembly is illustrated in Figure 1C. The study included the following groups: Group A, untreated defect; Group B, defect filled with cancellous bone; Group C, defect filled with cement; and Group D, defect treated with the cement-screw technique (Figures 1D–G).

### 2.3 Material properties, boundary and loading conditions

The fully assembled finite-element models for each group were imported into Abaqus 2022 (Simulia, Providence, RI, United States). Material properties were assigned as detailed in Table 1, with Poisson’s ratio and Young’s modulus based on parameters from previous research studies (Ren et al., 2022; Brihault et al., 2016; Chan et al., 2014). It is noteworthy that the finite-element model of the tibia used in this study has been validated in prior research (Ren et al., 2022). Then, assemble all components into a single entity and create a static analysis step. Given the strong adhesive properties of bone cement, interactions between components were defined as bonded. Since the intervention site was the bone defect on the medial tibial plateau, and considering that revision TKA is often necessitated by aseptic loosening near the cement-screw technique site, our focus was on evaluating the changes in the medial tibial plateau under various loading conditions (Berend et al., 2014). During normal gait, biomechanical loads on the knee joint are approximately two to three times body weight, with the medial and lateral plateau loads accounting for approximately 55% and 45% of the total load, respectively. Therefore, for a person weighing 65 kg, we selected loads of 350, 700, and 1050 N (equivalent to 1-, 2-, and 3-times BW) to simulate the loading on the medial aspect of the tibial plateau (Taylor et al., 2004; Zhao et al., 2007).

**TABLE 1:** Material properties.

Material	Young’s modulus (MPa)	Poisson’s ratio
Cortical bone	14,000	0.30
Cancellous bone	700	0.30
Tibia tray and stem	110,000	0.30
Bone cement	3,000	0.37
Screw	110,000	0.30

The applied loads were oriented parallel to the *Z*-axis of the model’s coordinate system. Additionally, the distal tibia was fixed by constraining all nodes at the distal end. Each component of the model was discretized using quadratic tetrahedral elements, with mesh sizes configured according to recommended standards. The total number of elements and nodes for each group model is summarized in Supplementary Table SI. Static analyses were conducted through finite-element simulations on the various models. The FEA methods and procedures employed were well-established and scientifically validated, as detailed in the relevant literature (Sun et al., 2022). We evaluated the von Mises stress distribution, maximum von Mises stress, and displacements induced under vertical compression in each component of the model.

### 2.4 Design and fabrication of a standardized surgical guide for guiding cutting and creating anteromedial tibial inclusive bone defects

**FIGURE 2:**
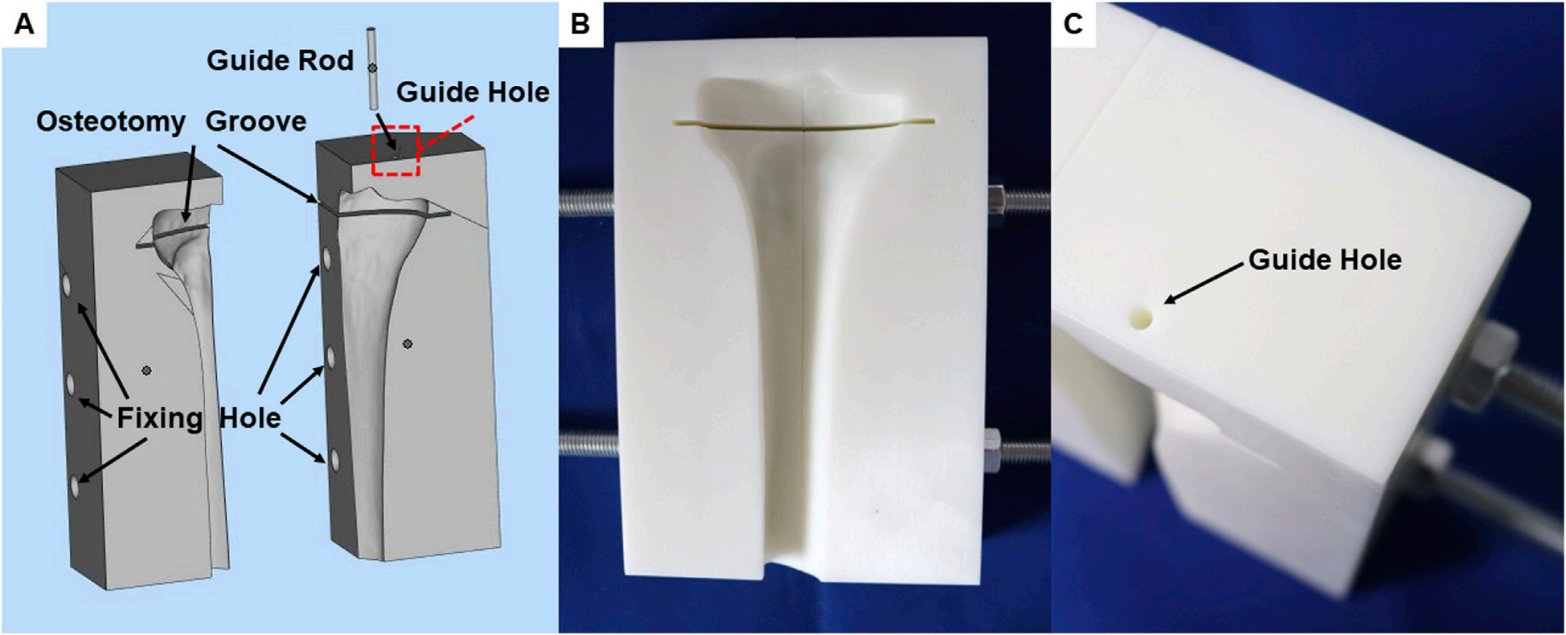
A unified surgical guide plate for biomechanical tests models. **(A)** Integral view of guide plate during 3D design. **(B)** Front view of the printed guide plate. **(C)** The position of the guide hole in the guide plate.

To ensure the accuracy and consistency of all biomechanical test models in this study, we first designed and 3D-printed a standardized guide for tibial plateau cutting and bone defect creation (Figure 2A). In alignment with the finite-element model, the surgical guide for tibial plateau cutting was positioned 8 mm below the highest point of the lateral joint surface and oriented perpendicular to the mechanical axis. The guide also incorporated a perforated hole above the medial anterior tibial plateau to facilitate the creation of a standardized circular bone defect. The dimensions of this guiding hole served as a reference for creating a standardized bone defect, ensuring precise drilling through the hole to the tibial plateau surface.

The guide plate was positioned 17 cm from the distal end of the tibial plateau cutting slot to ensure accurate distal tibial cutting when the saw blade was aligned with the distal plane of the guide plate. Additionally, threaded rod holes were integrated at both ends of the guide plate to secure fixation between the tibial model and the guide plate. For enhanced precision and rigidity, both the surgical guide and guiding pins were 3D-printed using resin material (Figures 2B, C).

### 2.5 Manufacturing of a bone defect guiding drill bit

To ensure compatibility with the finite-element model and consistency of the inclusive bone defects in the biomechanical test model, we designed and machined a tungsten steel drill bit with a 6 mm radius hemisphere and a depth of 6 mm (Supplementary Figures S1, S2A). Using this drill bit on the tibial plateau bone surface creates a bone defect that matches the shape and volume specified by the finite-element model (Supplementary Figures S2B, C).

### 2.6 Construction of biomechanical tests models

Twelve synthetic tibias (LS1365; Sybone AG, Switzerland) were utilized as the basis for the biomechanical test models. These synthetic tibial models, manufactured from a single production batch, demonstrate consistent material properties and geometric structures. Each model features a rigid foam cortical shell that is filled with cancellous bone material.

The synthetic tibias were randomly assigned to four groups, with each group comprising three specimens. The bone models were secured to the guide plate using threaded rods and nuts on either side. Tibial plateau cutting was carried out with an oscillating saw, commonly used in TKA surgery, through the cutting slot in the surgical guide plate. A guide pin, coated with ink, was inserted through the bone defect guide hole in the guide plate, leaving an ink mark on the bone surface. The grinding drill bit was then used to drill and grind centered on the ink mark, creating a standardized inclusive bone defect.

**FIGURE 3:**
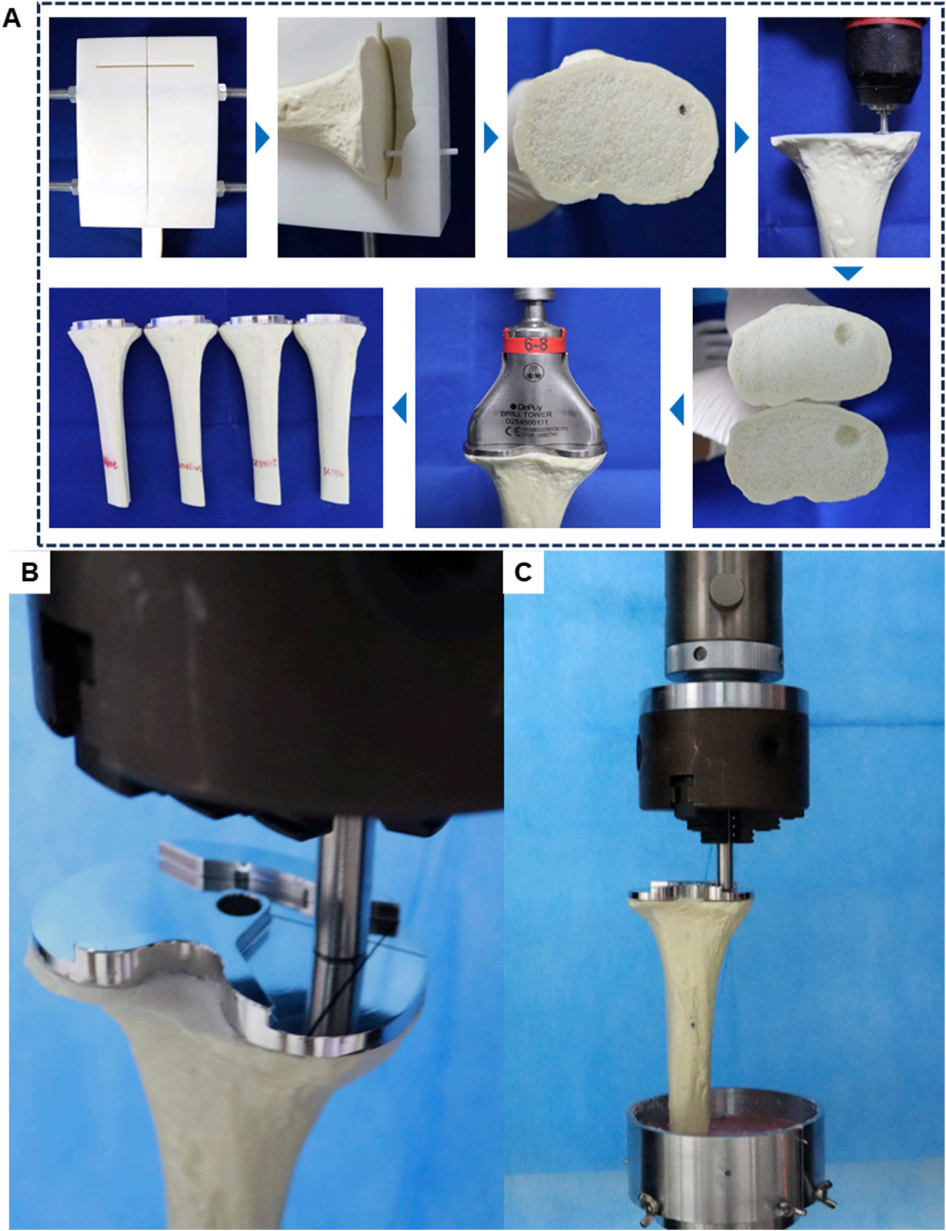
Schematic diagram of model assembly and experiment in biomechanical test. **(A)** Biomechanical test model manufacturing process. **(B)** Load compression position in biomechanical test. **(C)** General diagram of biomechanical test.

Group A received no specific treatment for the inclusive bone defect. Groups B, C, and D were treated with cancellous bone filling, cement filling, and a cement-screw technique, respectively. In group B, cancellous bone portions from other intact Synbones in the same batch were used to make cancellous bone blocks to ensure that the cancellous bone at the defect was the same as the surrounding cancellous bone material (Supplementary Figure S3). In Group D, a screw was implanted perpendicularly into the center of the bone defect using cancellous bone screws commonly used in TKA surgery, with dimensions matching those in the finite-element model. The tibial implant, best suited to the plateau, was selected and implanted following standard TKA procedures. Bone cement was evenly applied around the implant-bone interface and the screw areas of the defect. In addition, during the assembly process of each group of models, the use of bone cement around the bone defect was strictly reduced, so as to ensure that there was no bone cement in the bone defect of the no specific treatment group. The implant was then firmly seated in the tibia to ensure a solid connection between the implant, Sybone, and screw. After the bone cement had solidified, distal tibial cutting was performed with an oscillating saw along the guide plate’s distal end for subsequent biomechanical tests. The assembly process of the biomechanical test models is depicted in Figure 3A.

### 2.7 Biomechanical tests

During normal gait, tibial implants typically experience primarily axial forces. Therefore, for post-TKA tibial implants, a customized cylindrical indenter is used to apply axial loads to the medial aspect of the tibial implant. The base area of the cylinder is smaller than the plane area of the tibial implant. The indenter is pre-fixed to ensure consistent positioning of the models (Figure 3B). Subsequently, denture base powder is poured into the fixtures surrounding the model and allowed to fully solidify before conducting compression tests.

Axial compression tests were conducted using an electronic universal testing machine (ME50, Sunco Group, Shenzhen, China) on four groups of models (n = 3 per group). Each model was subjected to progressively increasing axial compression loads at a rate of 5 mm/min. Load-displacement data were recorded for each model (Figure 3C). Displacement measurements were taken at three axial load levels (350, 700, and 1050 N) to assess the biomechanical stability of the models.

### 2.8 Statistical analysis

Bone cyst sizes are presented as the mean ± standard error of the mean (SEM). Each biomechanical test group consisted of three replicate samples, all of which were successfully tested and included in the data analysis. Results are presented as mean values with standard deviation (SD). Statistical analysis was performed using GraphPad Prism 9.5. One-way analysis of variance (ANOVA) was used to determine significance. A *p*-value of < 0.05 was considered statistically significant.

## 3 Results

Analysis of X-ray data from 31 patients scheduled for TKA surgery, all of whom had preoperative X-rays showing tibial bone cysts, revealed that in 25 of these patients, the cysts were predominantly located in the anteromedial region of the tibial plateau (Table 2). Details of the sizes of the anteromedial bone cysts are provided in Table 3.

**TABLE 2:** Tibial plateau bone cyst location distribution.

Location	Anteromedial area	Medial middle area	Posteromedial area
The number of cases	25	4	2

**TABLE 3:** Tibial plateau anteromedial bone cyst size.

Direction	Vertical direction	Anteroposterior direction	Mediolateral direction
Size (mm)	8.1 ± 1.67	11.7 ± 1.94	11.3 ± 2.3

**FIGURE 4:**
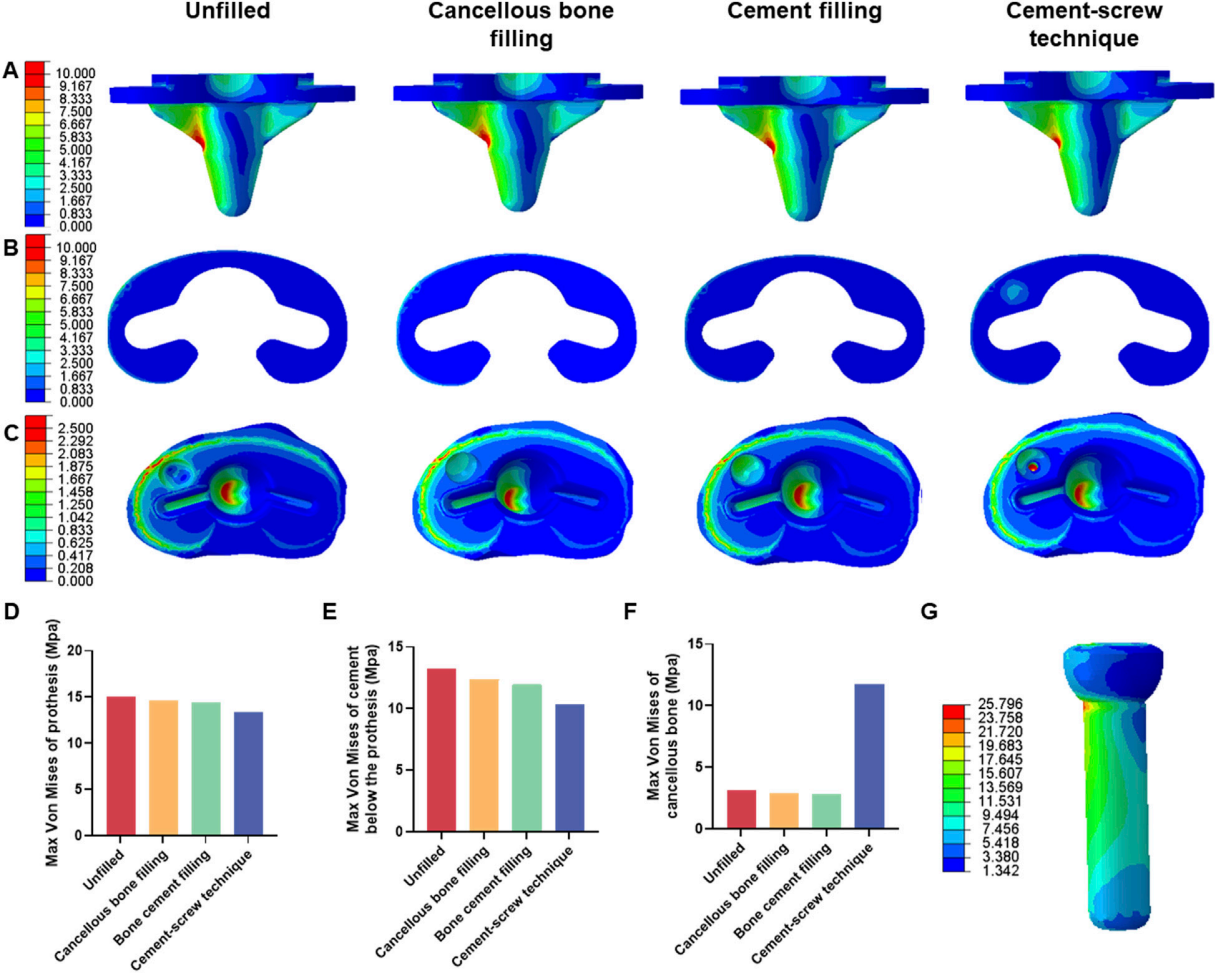
Von mises stress on each model component under 1050 N load. **(A)** Von mise stress on prothesis. **(B)** Von mise stress on cement below the prothesis. **(C)** Von mise stress on cancellous bone. **(D)** Bar graph of prothesis maximum stress. **(E)** Bar graph of cement below the prothesis maximum stress. **(F)** Bar graph of cancellous bone maximum stress. **(G)** Von mise stress on screw in cement-screw technique group.

FEA results of this study offer a detailed comparison of stress and displacement across different model groups. Under a load of 1050 N, the maximum von Mises stresses for the prosthesis in the four groups were as follows: Group A, 15.059 MPa; Group B, 14.631 MPa; Group C, 14.382 MPa; and Group D, 13.379 MPa (Table 4). Stress concentrations in the prosthesis’s high-stress regions were predominantly located on the medial side near the defect area, with no significant differences observed among the groups (Figure 4A). For the von Mises stresses in the cement below the prosthesis, the maximum values were: Group A, 13.255 MPa; Group B, 12.365 MPa; Group C, 11.926 MPa; and Group D, 10.321 MPa (Table 5). In Group D, a stress concentration zone was noted in the cement area above the screw, while other regions showed no significant differences in stress distribution (Figure 4B). The maximum von Mises stresses of the cancellous bone in the four groups were: Group A, 3.133 MPa; Group B, 2.919 MPa; Group C, 2.835 MPa; and Group D, 11.730 MPa (Table 6). In Group D, a stress concentration was observed near the screw insertion site. While high-stress concentrations were noted at the medial cancellous bone edges in Groups A, B, and C, the medial cancellous bone edges in Group D did not exhibit significant high-stress areas (Figure 4C). Group D showed the lowest maximum von Mises stresses in both the prosthesis and the cement below it, yet the highest maximum von Mises stresses in the cancellous bone. These variations are clearly depicted in the bar graphs (Figures 4D–F), with stress distributions following consistent trends under different loads (Tables 4–6). Additionally, the maximum von Mises stress of screw in Group D model was 25.796 MPa (Figure 4G).

**TABLE 4:** Max von Mises stress of the prothesis in each set of models.

Group	Max von Mises stress (MPa)		
	350 N	700 N	1050 N
A	5.020	10.039	15.059
B	4.877	9.754	14.631
C	4.794	9.588	14.382
D	4.460	8.920	13.379

**TABLE 5:** Max von Mises stress of the cement below the prothesis in each set of models.

Group	Max von Mises stress (MPa)		
	350 N	700 N	1050 N
A	4.418	8.837	13.255
B	4.122	8.243	12.365
C	3.975	7.951	11.926
D	3.440	7.000	10.321

**TABLE 6:** Max von Mises stress of cancellous bone in each set of models.

Group	Max von Mises stress (MPa)		
	350 N	700 N	1050 N
A	1.044	2.089	3.133
B	0.973	1.946	2.919
C	0.945	1.890	2.835
D	3.910	7.820	11.730

**FIGURE 5:**
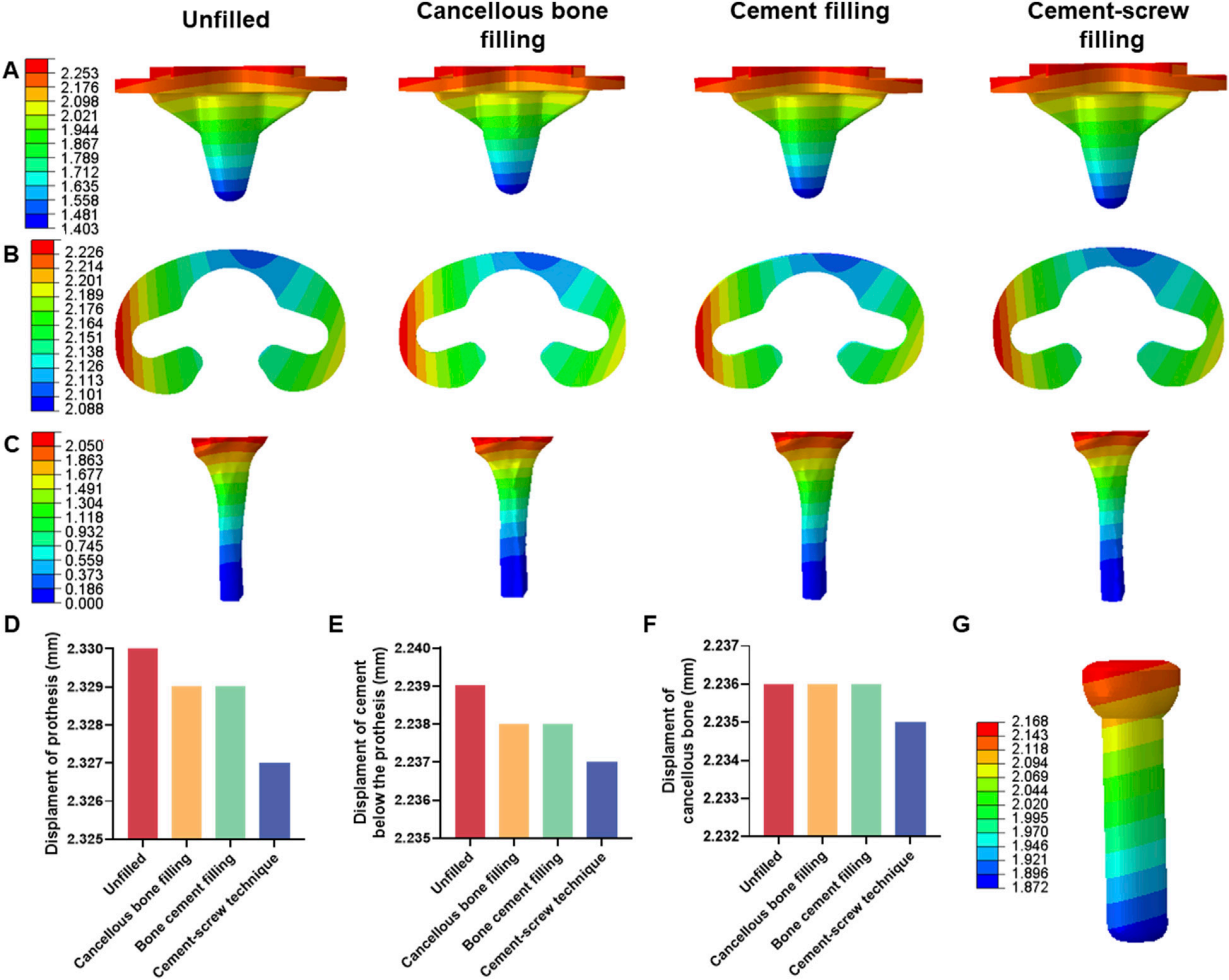
Displacement of each model under 1050 N load. **(A)** Displacement of prothesis. **(B)** Displacement of cement below the prothesis. **(C)** Displacement of cancellous bone. **(D)** Bar graph of prothesis maximum displacement. **(E)** Bar graph of cement below the prothesis maximum displacement. **(F)** Bar graph of cancellous bone maximum displacement. **(G)** Displacement of screw in cement-screw technique group.

Under a load of 1050 N, the maximum displacements of the prosthesis in Groups A, B, C, and D were 2.330 mm, 2.329 mm, 2.329 mm, and 2.327 mm, respectively (Supplementary Table SII). The maximum displacements of the cement below the prosthesis in these groups were 2.329 mm, 2.328 mm, 2.328 mm, and 2.327 mm, respectively (Supplementary Table SIII). The maximum displacements of the cancellous bone were 2.326 mm, 2.326 mm, 2.326 mm, and 2.325 mm, respectively (Supplementary Table SIV). There were no significant differences in displacement distributions among the various components (Figures 5A–C). Notably, Group D demonstrated the smallest displacements across all components (Figures 5D–F). This trend in displacement was consistent across different loading conditions (Supplementary Tables S2–S4). Additionally, the screw displacement in Group D models was 2.168 mm (Figure 5G).

**FIGURE 6:**
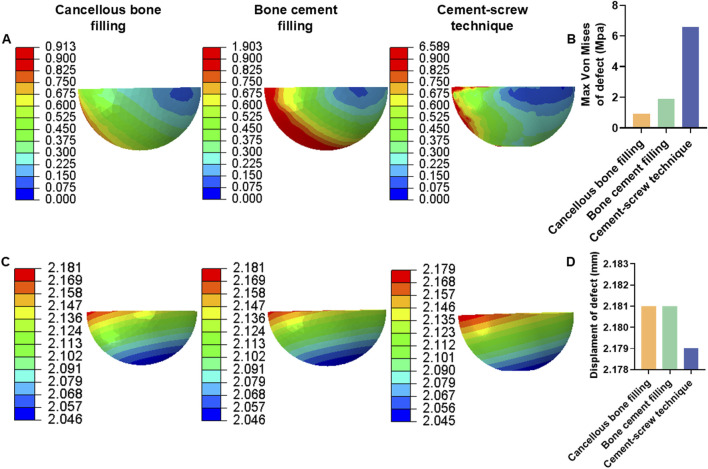
Von mises stress and displacement of defect area under 1050 N load. **(A)** Von mise stress of defect area. **(B)** Bar graph of defect area maximum stress. **(C)** Displacement of defect area. **(D)** Bar graph of defect area maximum displacement.

Additionally, under a load of 1050 N, in Group B, the maximum von Mises stress in the defect area filled with cancellous bone was 0.913 MPa, whereas Groups C and D showed maximum stresses of 1.903 MPa and 6.589 MPa, respectively, in the defect area filled with cement (Figure 6A). The maximum von Mises stress of the defect area in group D was the highest among the three groups (Figure 6B). Group B had a displacement of 2.181 mm in the defect area filled with cancellous bone, while Groups C and D showed displacements of 2.181 mm and 2.179 mm, respectively, in the defect areas filled with bone cement (Figure 6C). The displacement of the defect area in group D was the lowest among the three groups (Figure 6D).

**FIGURE 7:**
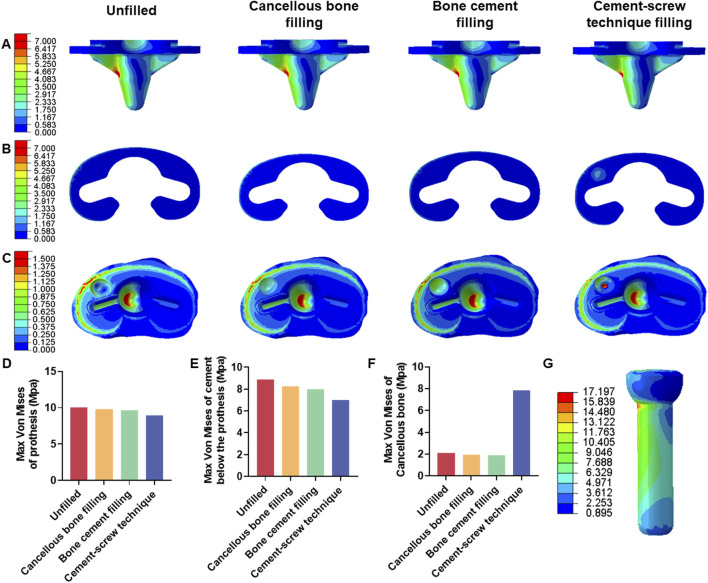
Von mises stress on each model component under 700 N load. **(A)** Von mise stress on prothesis. **(B)** Von mise stress on cement below the prothesis. **(C)** Von mise stress on cancellous bone. **(D)** Bar graph of prothesis maximum stress. **(E)** Bar graph of cement below the prothesis maximum stress. **(F)** Bar graph of cancellous bone maximum stress. **(G)** Von mise stress on screw in cement-screw technique group.

Under a load of 700 N, the study revealed that Group D exhibited the lowest maximum von Mises stress in the prosthesis, at 8.920 MPa, and in the cement below the prosthesis, at 7.000 MPa, compared to the other groups (Tables 4, 5). Conversely, Group D showed the highest maximum von Mises stress in the cancellous bone, reaching 7.820 MPa (Table 6). The stress distribution patterns among the groups at 700 N load were consistent with those observed at a 1050 N load (Figures 7A–C). These differences in maximum von Mises stress among components also align with observations at 1050 N and are clearly depicted in the bar graphs (Figures 7D–F). Additionally, the maximum von Mises stress in the screws of Group D models was 17.197 MPa (Figure 7G). Group B experienced a maximum von Mises stress of 0.609 MPa in the defect area filled with cancellous bone, while Groups C and D had maximum stresses of 1.269 MPa and 4.393 MPa, respectively, in the defect areas filled with cement (Supplementary Figure S4A). The maximum von Mises stress of the defect area in group D was the highest among the three groups (Supplementary Figure S4B).

**FIGURE 8:**
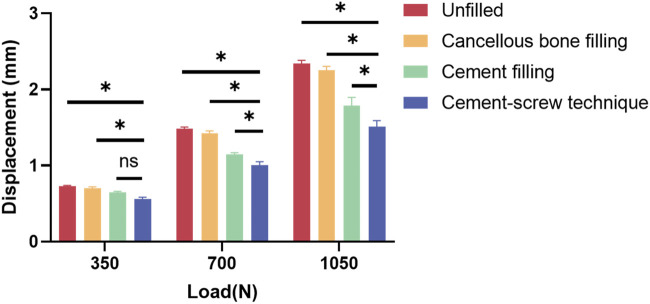
Displacement results of static compression tests. Data are shown as mean ± SD. *P < 0.05 and ns = not significant.

Table 7 displays the vertical displacements of each model under static compression tests with three different axial loads. The cement-screw technique group demonstrated the smallest displacements. Notably, at axial loads of 700 N and 1050 N, the displacements in the cement-screw technique group were significantly smaller compared to the other three groups, with statistically significant differences (*p* < 0.05). Even at a load of 350 N, the displacement in the cement-screw technique group was significantly smaller than that in both the untreated group and the cancellous bone filling group (*p* < 0.05) (Figure 8).

**TABLE 7:** Vertical displacement of the models in the static test.

Group	Displacement (mm)		
	350 N	700 N	1050 N
Unfilled	0.74/0.72/0.72	1.51/1.47/1.47	2.39/2.32/2.32
Cancellous bone filling	0.70/0.68/0.72	1.46/1.40/1.41	2.31/2.22/2.24
Cement filling	0.64/0.66/0.65	1.13/1.17/1.15	1.71/1.91/1.75
Cement-screw technique	0.57/0.54/0.58	1.02/0.95/1.04	1.53/1.42/1.58

## 4 Discussion

During TKA, residual tibial bone defects resulting from tibial bone cysts can significantly impact the reconstruction of knee joint anatomy and function (Lombardi et al., 2010; Cuckler, 2004; Bush et al., 2006). Consequently, managing tibial bone defects presents a technical challenge in TKA. A single tibial bone defect is classified as AORI Type 2A according to the Anderson Orthopaedic Research Institute (AORI) classification system (Sheth et al., 2017). Techniques such as bone filling, bone cement filling, and cement-screw technique are employed to address AORI Type 2A tibial bone defect. However, there is no consensus on which technique provides the optimal postoperative outcomes in TKA. Currently, the selection of technical parameters is often guided by the practices of experienced surgeons or personal experience, rather than robust evidence. In the realm of modern precision surgery, advancements in the accuracy and biomechanical stability of managing bone defects resulting from bone cysts are anticipated (Bargar, 2007; Chi et al., 2014). This study aims to compare the efficacy of these three techniques against no treatment for managing anteromedial tibial bone defect during TKA.

Bone filling can be utilized for defects of various sizes and shapes, effectively restoring bone volume. However, it carries risks such as prolonged surgical time, nonunion, delayed healing, disease transmission, infection, and graft resorption (Lotke et al., 2006; Bauman et al., 2009; Wilke et al., 2019). Bone cement filling and the cement-screw technique are also applicable for defects of differing sizes and shapes (Berend et al., 2014; Panegrossi et al., 2014). These methods not only fill the defects but also adhere to the surrounding tissues. Additionally, the implantation of screws in the cement-screw technique can further enhance stability at the defect site.

In the methodology section, we developed a representative tibial bone defect model based on clinical cases of tibial bone cysts and integrated the advantages of FEA with biomechanical test to address the limitations inherent in each method when used in isolation. To overcome the challenges posed by variability in traditional experimental models, we innovatively designed and 3D-printed surgical guides to produce customized and standardized cutting models. Given that the average depth of the tibial anteromedial bone cysts in our clinical cases is approximately 8 mm and that the typical resection depth of the medial tibial plateau during clinical tibial plateau cutting is about 2 mm, we set the tibial bone defect depth in our finite-element model to 6 mm. Additionally, based on the average anteroposterior and mediolateral dimensions of the bone cysts from clinical data, and to ensure design accuracy and applicability, we designed a hemispherical drill bit with a 6 mm radius to create standardized defects. We further simulated stress distribution and displacement for each model under three different loading conditions using FEA. Static compression tests were then conducted under these three different loads to replicate knee joint loading scenarios and measure model displacement.

The biomechanical properties of implants are crucial for their stability (Bori et al., 2022; Apostolopoulos et al., 2022). An effective approach to defect management not only reduce stress of the surrounding components but also facilitates efficient transfer of stress from the proximal tibia to the distal tibia, thereby reducing the risk of periprosthetic fractures and implant loosening (Lescun et al., 2020; Khan et al., 2016). Our FEA results show that in the cement-screw group, the maximum stress in both the tibial prosthesis and the cement below it is minimized. Additionally, the maximum stress in the cancellous bone and the defect area is maximized, with the smallest area of high stress concentration observed at the medial platform of the cancellous bone. These findings indicate that the cement-screw technique effectively distributes stress across the tibial prosthesis and surrounding components, while also efficiently transferring stress from the proximal to the distal tibia.

In the untreated group, the lack of cancellous bone on the tibial plateau hinders the transfer of stress from the defect area to the distal region. Conversely, when the defect is filled with cancellous bone or bone cement, the intact cancellous bone surface serves as an intermediary for load transfer, promoting stress distribution to the distal region. The use of high modulus of elasticity screws combined with cement offers improved support and stability to the defect area, thereby enhancing the efficiency of load transfer to the distal regions (Siddiqi et al., 2021). According to Wolff’s Law, bone structures adapt to resist applied forces, with bone mass decreasing under excessively low stress (Teichtahl et al., 2015; Wang et al., 2021; Barak et al., 2011). Therefore, effective load transfer can help maintain adequate bone volume under the prosthesis following TKA.

Although FEA revealed no significant differences in component displacements between the cement-screw group and the other three groups, static compression tests highlighted the distinctions among the models. In all four models, the cement-screw group demonstrated the smallest displacements under all three loading conditions, with significant differences observed at 700 N and 1050 N loads compared to the other groups. These findings emphasize the alignment between FEA and biomechanical tests, confirming that the cement-screw technique provides superior biomechanical performance.

This study has several limitations. First, we used synthetic bone rather than cadaveric bone for biomechanical tests. However, synthetic bone offers standardized dimensions and properties, and can be used with standardized bone defect models and drill bit to ensure consistency between experimental samples and accuracy of results. Second, tibial defects in clinical practice vary widely, and our study focused on only one type of inclusive defect. However, the bone defect model used is representative of anteromedial inclusive defect on the tibial plateau resulting from bone cysts encountered in recent TKA surgeries. Despite these limitations, our study offers valuable methodological insights for future research on managing various types and locations of bone defects.

## 5 Conclusion

In summary, our study highlights the effectiveness of using cement-screw technique to repair residual tibial bone defects from bone cysts during TKA. This approach effectively reduces Von Mises stress in surrounding components and minimizes model displacement. Therefore, to optimize the biomechanical strength of the prosthesis and improve implant survival rates, we recommend adopting cement-screw technique for managing inclusive tibial bone defect following tibial plateau bone cyst in TKA.

## Data Availability

The original contributions presented in the study are included in the article/Supplementary Material, further inquiries can be directed to the corresponding authors.
