# Supplementary material for: Biomechanical analysis of different techniques for residual bone defect from tibial plateau bone cyst in total knee arthroplasty
Source: Front Bioeng Biotechnol. 2024 Oct 30;12:1498882. doi: 10.3389/fbioe.2024.1498882 (PMC11557455; doi:10.3389/fbioe.2024.1498882)
Supplement: Supplementary file 1 [file Table1.DOCX]

Biomechanical Analysis of Different Techniques for Residual Bone Defects from Tibial Plateau Bone Cysts in Total Knee Arthroplasty

**Dehua Liu, MD^1†^, Zhuang Miao, PhD^1,2†^, Wenfei Zhang, MD^3^, Chuanwen Liu, MD^4^, Longzhuo Du, MD^1^, Yuanlong Zhu, BS^2^, Yange Luo, MD^1^ , Weibo Zheng, MD^1^, Jianli Zhou, MD^5^, Peilai Liu, PhD^1^ *, Xuezhou Li^1^ *, Ming Li,PhD^4^ ***

†Dehua Liu, and Zhuang Miao contributed equally to this work and share first authorship.

* Corresponding author.

^1^ Department of Orthopaedics, Qilu Hospital, Cheeloo College of Medicine, Shandong University, Jinan, Shandong, 250012, China.

^2^ Key Laboratory of Ultra-Weak Magnetic Field Measurement Technology, Ministry of Education, School of Instrumentation and Optoelectronic Engineering, Beihang University, Beijing 100191, People's Republic of China.

^3^ Psychological department, Qilu Hospital of Shandong University Dezhou Hospital, Dezhou, Shandong, 253000, China.

^4^ Department of Orthopaedics, Qilu Hospital of Shandong University Dezhou Hospital, Dezhou, Shandong, 253000, China.

^5^ Nuclear medicine department, Qilu Hospital of Shandong University Dezhou Hospital, Dezhou, Shandong, 253000, China.

***Correspondence:**Ming Li, Department of Orthopaedics, Qilu Hospital of Shandong University Dezhou Hospital, Dezhou, Shandong, 253000, China. E-mail: [gklm1628@163.com](mailto:gklm1628@163.com)

Xuezhou Li, Department of Orthopaedics, Qilu Hospital of Shandong University, Jinan, 250012, China. E-mail: [lixuezhou@sdu.edu.cn](mailto:lixuezhou@sdu.edu.cn)

Peilai Liu, Department of Orthopaedics, Qilu Hospital of Shandong University, Jinan, 250012, China.

E-mail: [199362000205@emailsdu.edu.cn](mailto:199362000205@emailsdu.edu.cn)

| **Table. Sup****I. Number of nodes and elements for the four models.** | | |
| --- | --- | --- |
| **Model** | **Nodes** | **Elements** |
| Group A | 441734 | 287099 |
| Group B | 443979 | 288451 |
| Group C | 443979 | 288451 |
| Group D | 480411 | 312535 |

| **Table. SupII. Displacement of the prothesis in each set of models.** | | | |
| --- | --- | --- | --- |
| **Group** | **Displacement (mm)** | | |
|  | **350N** | **700N** | **1050N** |
| A | 0.777 | 1.553 | 2.330 |
| B | 0.777 | 1.553 | 2.329 |
| C | 0.776 | 1.553 | 2.329 |
| D | 0.776 | 1.552 | 2.327 |

| **Table. SupIII. Displacement of the cement below the prothesis in each set of models.** | | | |
| --- | --- | --- | --- |
| **Group** | **Displacement (mm)** | | |
|  | **350N** | **700N** | **1050N** |
| A | 0.746 | 1.493 | 2.239 |
| B | 0.746 | 1.492 | 2.238 |
| C | 0.746 | 1.492 | 2.238 |
| D | 0.746 | 1.491 | 2.237 |

| **Table. SupIV. Displacement of cancellous bone in each set of models.** | | | |
| --- | --- | --- | --- |
| **Group** | **Displacement (mm)** | | |
|  | **350N** | **700N** | **1050N** |
| A | 0.745 | 1.491 | 2.236 |
| B | 0.745 | 1.491 | 2.236 |
| C | 0.745 | 1.490 | 2.236 |
| D | 0.745 | 1.490 | 2.235 |


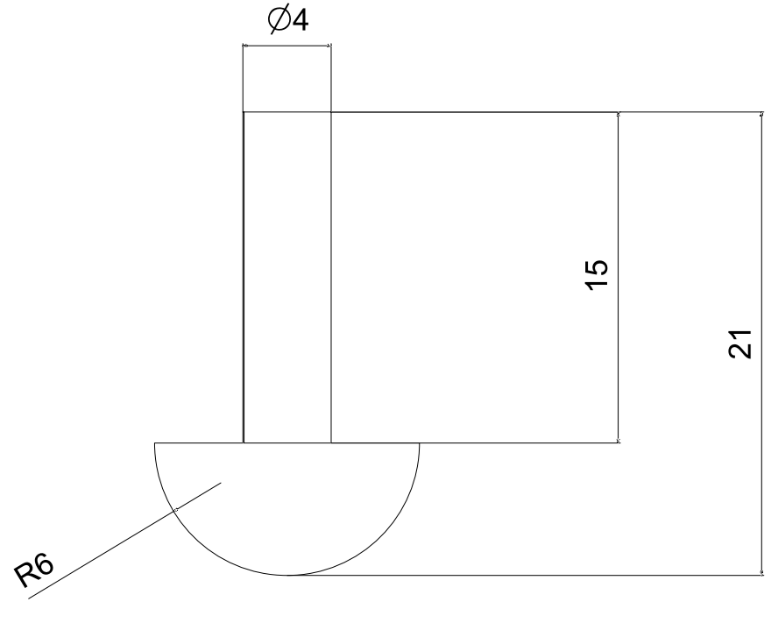


**Figure. Sup1.** (A) Design drawing of the drill bit.


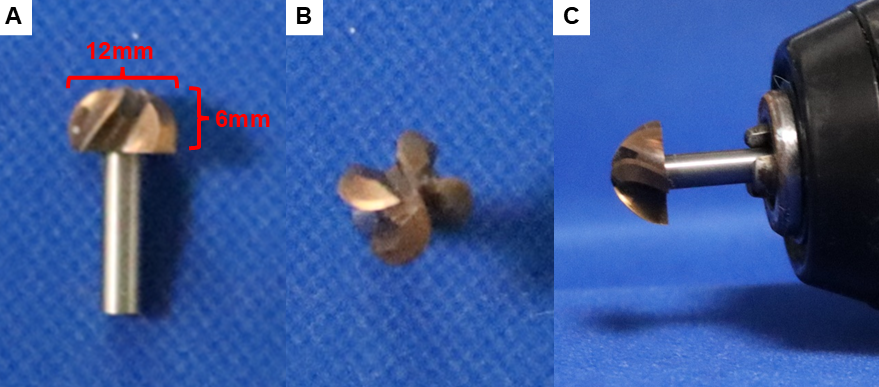


**Figure. Sup2.** A drill bit for biomechanical tests models. (A) Specific parameters of the drill bit. (B) Superior view of the drill bit. (C) Lateral view of the drill bit.


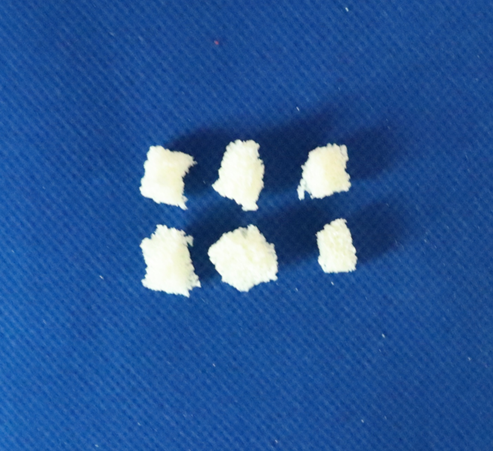


**Figure. S3.** Cancellous bone filling material.


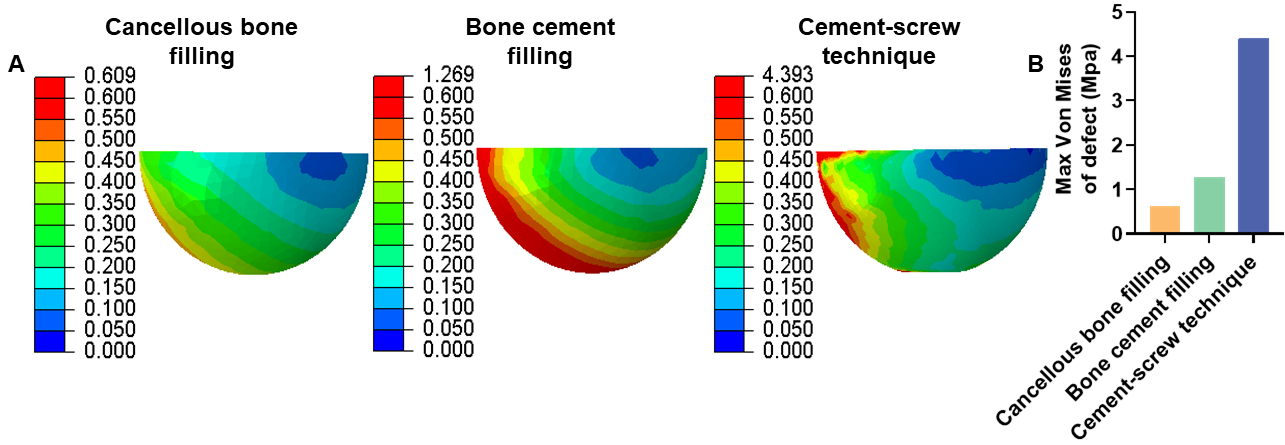


**Figure. S4.** Von mise stress on defect under 700 N load. (A) Von mise stress on defect. (B) Bar graph of defect maximum stress.
